# Conditions Simulating Enlargement of the Liver.—I

**Published:** 1909-05-15

**Authors:** 


### CONDITIONS SIMULATING ENLARGEMENT OF THE LIVER.—I.

Enlargement of the liver may be simulated if the organ is displaced downward or if it is deformed. It may be displaced downwards by the following conditions: Emphysema; pleuritic effusion (right side); empyema (right side); pneumothorax (right side); pericardial effusion; subdiaphragmatic abscess; hepatoptosis. It may be deformed: congenitally or by tight lacing.

In emphysema the physical signs and the altered shape of the chest would be apparent. The difficulty arises when emphysema, with or without bronchitis, causes symptoms of failure of the right side of the heart: is the liver that may be palpable in such a case merely displaced downwards, or is it also enlarged from nutmeg change?

Emphysema is prone to occur in alcoholic subjects, and difficulty arises in distinguishing a cirrhotic from a merely displaced organ. The hardness in the cirrhotic case, the tenderness when there is recent nutmeg change, help considerably in the diagnosis.

In pleuritic effusion or in empyema of the right side the liver may be pushed down and the edge felt below the costal margin. Dulness at the right base might, of course, be due to a large tumour arising from the upper part of the right lobe, or to a hepatic abscess, or to a subdiaphragmatic abscess. One of the ways of distinguishing between these and an effusion into the right pleural cavity is to be very careful in the percussion of the dulness behind, in the axilla, and in front, with a view to marking the outline of its upper limit with all the accuracy possible, that is.

In cases of pleuritic effusion the dulness as a rule extends higher behind than it does in the axilla, and higher in the axilla than it does in front; whereas when due to subdiaphragmatic or hepatic causes it is quite often higher in the axilla than it is either behind or in front; in other words, the outline of the dulness is dome-shaped, with the convexity of the dome highest in the axilla. In cases of pneumothorax the physical signs are nearly always sufficiently distinctive.

In all these conditions an important point is that the organ will be found to move very little, if at all, during respiration, or even as the result of a maximum inspiratory effort.

Subdiaphragmatic abscess is frequently obscure and difficult to diagnose. It is apt to be mistaken for empyema or for hepatic abscess. When a subdiaphragmatic abscess has arisen as the result of perforating gastric or duodenal ulcer, the physical signs may be particularly baffling, owing to the fact that the abscess cavity is likely to contain gas as well as pus; pyopneumothorax may be simulated.

Dislocation and undue mobility of the liver may result from the great weight of a much enlarged organ, the actual increase in size being thereby exaggerated. On the other hand, as part of a general enteroptosis, a perfectly normal-sized liver may come so low in the abdomen that it may mislead the observer into regarding it as increased in size. Hepatoptosis is most marked when the patient is in the erect position. A large part of the surface of the viscus comes into contact with the abdominal wall, and in some cases the dislocation is so great that on palpation the hand can be placed between its upper border and the costal margin. If hepatoptosis is suspected the abdomen should be examined both when the patient is supine and also when standing.

A curious form of displacement which may simulate enlargement is tilting of the right lobe downwards and inwards so that the normal transverse axis of the organ becomes vertical, and the edge, instead of being obliquely transverse, may be lying almost vertically, with the tip of the gall bladder pointing to the left instead of downwards.

The "tight-lacing" liver is usually much elongated in its vertical axis, the lateral pressure of tight lacing, as it were, squeezing out a portion from under the ribs. The edge of this projecting portion may reach to the level of the umbilicus or even to the iliac crest in the right nipple line without the volume and weight of the organ being greater than normal. Between the constricted portion and the upper and main part of the viscus there may be a deep transverse groove which comes down during inspiration so as to be palpable. The lower portion may be freely movable, and in consequence be mistaken for a dilated gall-bladder or for a movable kidney.

The sharp, well-defined lower edge should at once serve to distinguish it from a renal tumour; besides, on bimanual palpation it cannot be felt to fill out the loin in the way that a renal tumour nearly always does. From a true enlargement of the liver it must be distinguished by its limited size, its mobility, the detection of a transverse groove between it and the main part of the organ, its occurrence in women, and the general condition of the figure brought about by tight-lacing.

*(To be continued.)*

